# Brain mapping in cognitive disorders: a multidisciplinary approach to learning the tools and applications of functional neuroimaging

**DOI:** 10.1186/1472-6920-7-39

**Published:** 2007-10-22

**Authors:** Daniel J Kelley, Sterling C Johnson

**Affiliations:** 1 Geriatric Research Education and Clinical Center, William S. Middleton Memorial Veteran Hospital, Madison, WI, USA; 2 Department of Medicine, University of Wisconsin School of Medicine and Public Health, Madison, WI, USA; 3 Waisman Laboratory for Brain Imaging and Behavior, Waisman Center, University of Wisconsin, Madison, WI, USA; 4 Neuroscience Training Program, Center for Neuroscience, University of Wisconsin School of Medicine and Public Health, Madison, WI, USA; 5 Medical Scientist Training Program, University of Wisconsin School of Medicine and Public Health, Madison, WI, USA; 6 HHMI Teaching Fellows Program, Wisconsin Program for Scientific Teaching, Department of Bacteriology, University of Wisconsin, Madison, WI, USA

## Abstract

**Background:**

With rapid advances in functional imaging methods, human studies that feature functional neuroimaging techniques are increasing exponentially and have opened a vast arena of new possibilities for understanding brain function and improving the care of patients with cognitive disorders in the clinical setting. There is a growing need for medical centers to offer clinically relevant functional neuroimaging courses that emphasize the multifaceted and multidisciplinary nature of this field. In this paper, we describe the implementation of a functional neuroimaging course focusing on cognitive disorders that might serve as a model for other medical centers. We identify key components of an active learning course design that impact student learning gains in methods and issues pertaining to functional neuroimaging that deserve consideration when optimizing the medical neuroimaging curriculum.

**Methods:**

Learning gains associated with the course were assessed using polychoric correlation analysis of responses to the SALG (Student Assessment of Learning Gains) instrument.

**Results:**

Student gains in the functional neuroimaging of cognition as assessed by the SALG instrument were strongly associated with several aspects of the course design.

**Conclusion:**

Our implementation of a multidisciplinary and active learning functional neuroimaging course produced positive learning outcomes. Inquiry-based learning activities and an online learning environment contributed positively to reported gains. This functional neuroimaging course design may serve as a useful model for other medical centers.

## Background

A need in medical education is to inform students about the application of a continuum of multidisciplinary imaging techniques to understand disease and to encourage physicians to take part in clinical imaging trials [1]. Higher level brain functions, such as memory, metacognition, executive abilities, language, and emotions, form the basis of adaptive and rich social interactions and their dysfunction can now be better understood through experimentation and application of advanced functional imaging techniques. With statistical analyses of images collected using functional imaging modalities, clinicians and researchers have the capability to spatially localize significant brain activation in the form of functional brain maps that convey information about the neural systems subserving aspects of cognition. Functional brain mapping topics have yet to be incorporated into the curriculum at most medical centers even though these techniques have demonstrated great clinical potential for advancing medical practice by informing physicians about the function of scanned brain structures [2-5]. This is in contrast to radiological neuroimaging techniques like CT and MRI that are incorporated into the medical curriculum [6] and are readily used to provide information about gross structural anatomy and pathology in clinical diagnosis. In order to understand the distinctive features of structural and functional imaging methodologies and to catalyze the translation of functional neuroimaging techniques from the research laboratory into clinical applications for patient care, physicians require a practical understanding of the entire functional imaging process from experimental design to the interpretation of statistical brain activation maps [7].

Brain mapping has great potential to influence medicine by serving as a biomarker for cognitive disorders that has utility in predicting or detecting disease and in quantifying the effect of therapeutic intervention [8]. For example, longitudinal studies of people at risk for Alzheimer's Disease may identify patterns of functional brain networks that are predictive of disease susceptibility. In addition, brain mapping studies may influence medical education by providing a rapidly growing knowledge-base of functional neuroanatomical relationships. However, to actualize the potential of brain mapping in medicine, practitioners must be educated in both the benefits and limitations of brain mapping in order to adequately interpret prior studies, take part in ongoing studies, and implement new studies.

The teaching of functional neuroimaging requires a multidisciplinary approach based on (1) the variety of functional imaging modalities used in basic and clinical neuroscientific investigations and (2) the multidisciplinary nature of the imaging sciences which requires expertise in neuroscience, neuropsychology, clinical neurology, neuroradiology, statistics, medical physics, computer science, and neuroanatomy, to name a few [9]. In the Fall 2006 course at the University of Wisconsin-Madison entitled, "Functional Imaging of Cognitive Disorders," we implemented a multidisciplinary, active learning course design which focused on cutting edge brain mapping methods that are used to measure structure and function in normal and previously normal brains and that, in particular, have demonstrated relevance to the assessment of brain function and localization in clinically defined cognitive disorders. Course design reflected the multidisciplinary approach used to brain map cognitive disorders and was weighted toward formal lectures on a variety of key topics regarding methodology and clinical applications, as well as student presentations and group discussions of seminal and current relevant scientific papers. The topics for neuroimaging techniques and cognitive disorders reflected the multidisciplinary nature of brain mapping in clinical research and patient case studies.

A complementary view to the idea that neuroimaging research holds great promise for improving classroom education [10-14] is that inquiries into the educational process of students may improve the teaching of functional neuroimaging. To assess the impact that our course design had on student gains in learning functional neuroimaging, henceforth referred to as neuroimaging cognition, we implemented the SALG [15] instrument. The SALG instrument was developed by Elaine Seymour at the University of Wisconsin Center for Educational Research to assess student perception of learning gains as a function of course design and delivery [16] and has been successfully used to this end [17,18]. We converted the online SALG instrument to a paper format and assessed student learning gains as part of our end of semester course assessment.

## Methods

### Course Design

The inquiry-based, active learning methods [19,20] used to engage students depended on regular attendance and active student participation. Activities included oral presentations, focused group discussions, and reading assessments which were developed by students. In addition, we assigned readings from textbooks [21,22] to provide an overview of the techniques and their applications. Learn@UW [23], an online learning environment, was used to deliver topics that included all research papers, case studies, reading assessments, solutions, lecture notes, and grades to students. Students were required to prepare for each class by reading the assigned papers and by completing reading assessments which their classmates constructed.

Each student was asked to present one paper in detail including the theory supporting it, the methods, results, and interpretation. To account for the diversity of student interests, students presented papers on topics which they selected and were approved by the instructor. Students were expected to prepare a 15 minute presentation and critique of the paper. The presentation was followed by an interactive period of commentary and discussion from the faculty and students. The presentations were assessed on clarity of content, scientific accuracy, and presentation quality. The presenting student was expected to consult several other sources beyond the paper (such as reviews and other empirical studies with similar or different methods) in order to develop a broader perspective on the topic, place the current paper in the context of what is known and not known about the topic, and to provide ideas for further study. In addition, one week prior to their presentation, students were asked to submit two questions (and answers) about the key concepts in the paper they were going to present which, upon approval, were forwarded to the class and posted on Learn@UW as reading assessments. Reading assessments consisted of responses to two cluster-style questions per week and were graded for content based on the assigned readings for that week. Course assessment was based on active class participation, individual student presentations, and weekly reading assessments

A vital component of the course was faculty lectures. Each week a presentation was given to students by UW faculty who are experts in their field. This was followed by a group discussion period in which students could ask questions and instructors engaged students through decision making questions.

We based topic selection on the advanced neuroimaging techniques currently in use and the expertise of faculty on the University of Wisconsin campus [see Additional file 1]. The neuroimaging techniques we selected for this course included: Magnetic Resonance Imaging (MRI), functional MRI (fMRI), Positron Emission Tomography, Single Photon Emission Computed Tomography, Transcranial Magnetic Stimulation, Electroencephalography, Diffusion Tensor Imaging, Diffusion Weighted Imaging, Perfusion Imaging, and Connectivity Techniques. The focus of clinical neuroimaging applications included: Alzheimer's Disease, Parkinson's Disease, Stroke, fMRI Presurgical Mapping, Anxiety, Depression, Pain, Traumatic Brain Injury, Social Cognition, and Cerebral Recovery. Guest lecturers were recruited from the University of Wisconsin Departments of Medicine, Radiology, Neurology, Medical Physics, Psychiatry, Psychology, Biomedical Engineering, Kinesiology, the Medical Scientist Training Program, and the Neuroscience Training Program.

### Survey and Statistical Analysis

Students voluntarily completed our implementation of the Student Assessment of Learning Gains (SALG), which was administered to assess student learning and gain feedback as part of the initial course evaluation. The questionnaire was voluntary and anonymous with no bearing whatsoever on the students' grades. Because the survey was part of the academic evaluation of the course, IRB approval was initially not required and not obtained. However, prior to publishing the material in this report, we received IRB approval for this secondary use of the data.

Online course access was determined using the number of topics accessed feature in Learn@UW. Responses to the SALG were anonymous and quantified by someone not affiliated with the course in the Neuroscience Training Program. Categorical responses were coded on a 5 point (0 = Not applicable; 1 = Not At All, 2 = A Little, 3 = Somewhat, 4 = A Lot, 5 = A Great Deal) or 4 point (0 = Not Applicable; 1 = Strongly Agree, 2 = Disagree, 3 = Agree, 4 = Strongly Agree) Likert scale. Questions in the neuroimaging cognition category focused on the gains in learning goals for this course and the course design questions were centered around engagement activities and course content. The polychoric correlation, rho, is a useful statistic to understand associations in categorical data and is preferred to the Spearman correlation because the discretizing latent variable thresholds are estimated [24]. The association of course contributions to neuroimaging cognition learning gains using the SALG instrument were determined using polychoric [25] correlation software in R [26]. Two-tailed significance was assessed after a rho to t conversion on N-2 degrees of freedom. A corrected p-value <= 0.05 was considered significant.

## Results

**Figure 1:**
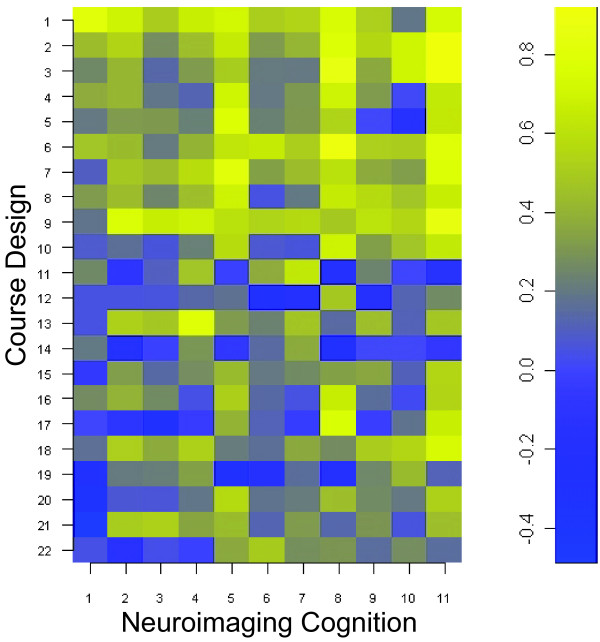
Polychoric Correlation Matrix of SALG Associations between Course Design and Neuroimaging Cognition.

**Figure 2:**
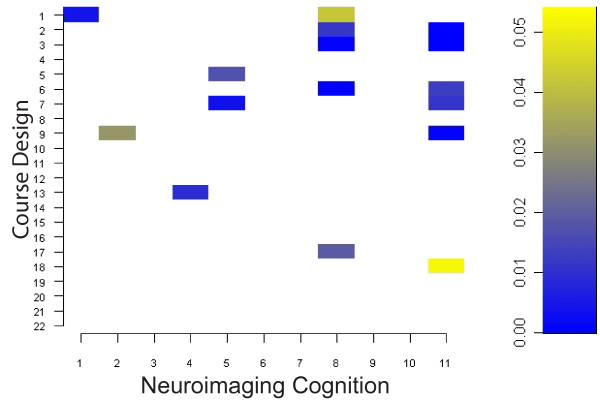
P-value Matrix of Significant (p <= 0.05) Polychoric Correlations in Figure 1, Bonferroni corrected for multiple comparisons.

Of all the students (N = 20) attending the course, ten were advanced undergraduates and the rest were professional students in graduate or medical school. All students completed the survey. Students accessed 94 +/- 3 percent of the course topics listed in the Learn@UW course website. Student responses to the SALG questions focusing on student neuroimaging cognition and course design are reported in Table 1 as means (+/- SD) and percentage frequency of Likert scaled responses. The polychoric correlation matrix (Figure 1) displays the association of student gains in medical neuroimaging cognition with course design. Significant association (p <= 0.05, 2-tailed) are plotted in the p-value matrix of Figure 2 and listed in Table 2. A marginally significant association was also found in learning gains between guest lecturers and future neuroimaging courses using the same format (rho = 0.73, df = 18, p = 0.07).

**Table 1:** SALG Questionnaire Results Reporting Mean, Standard Deviation, and Percentage of Responses in each Likert-scaled Category (N = 20).

	**Neuroimaging Cognition**	**Mean**	**SD**	**0**	**1**	**2**	**3**	**4**	**5**
Q1	Functional imaging patients with cognitive disorders can improve our understanding of basic neuroscience/psychology (i.e., normal brain function)	4.45	0.51	0%	0%	0%	0%	55%	45%
Q2	Understanding that future patients will benefit from ongoing neuroimaging research	4.15	0.81	0%	0%	5%	10%	50%	35%
Q3	Understanding that neuroimaging can improve the diagnosis of cognitive disorders	4.15	0.88	0%	0%	5%	15%	40%	40%
Q4	Understanding how neuroimaging techniques can impact medicine	4.05	1.00	0%	5%	0%	15%	45%	35%
Q5	Functional imaging can benefit patients with cognitive disorders	3.85	0.93	0%	0%	10%	20%	45%	25%
Q6	Confidence in your ability to take part in this field	3.85	0.93	0%	0%	5%	35%	30%	30%
Q7	Understanding that neuroimaging can improve the treatment of cognitive disorders	3.80	0.95	0%	0%	10%	25%	40%	25%
Q8	Enthusiasm for this field	3.80	1.06	0%	0%	10%	35%	20%	35%
Q9	Understanding that neuroimaging can improve the prognosis of cognitive disorders	3.75	1.07	0%	5%	5%	25%	40%	25%
Q10	Neuroimaging should be an integral part of medical education	3.50	0.61	0%	0%	5%	40%	55%	-
Q11	Future neuroimaging courses should use the same format as this course	3.05	0.69	0%	5%	5%	70%	20%	-
									
	**Course Design**								
Q1	Guest Lectures	4.40	0.60	0%	0%	0%	5%	50%	45%
Q2	The Topics Covered	4.35	0.81	0%	0%	5%	5%	40%	50%
Q3	Overall Course	4.35	0.81	0%	0%	5%	5%	40%	50%
Q4	Neuroimaging Techniques Overall	4.20	0.89	0%	0%	0%	30%	20%	50%
Q5	Functional MRI	4.15	0.81	0%	0%	5%	10%	50%	35%
Q6	Importance of this field	4.15	0.81	0%	0%	0%	25%	35%	40%
Q7	Critically Reviewing Articles	4.10	0.79	0%	0%	5%	10%	55%	30%
Q8	Selected Papers	4.10	0.91	0%	0%	5%	20%	35%	40%
Q9	Summarizing Articles	3.95	0.94	0%	0%	5%	30%	30%	35%
Q10	Giving an Oral Presentation (neuroimaging skills)	3.80	0.95	0%	5%	0%	25%	50%	20%
Q11	Learn@UW was easy to use	3.70	0.47	0%	0%	0%	30%	70%	-
Q12	The overall content of the course was appropriate	3.65	0.49	0%	0%	0%	35%	65%	-
Q13	Answering Reading Assessments	3.65	1.23	5%	5%	0%	15%	60%	15%
Q14	Learn@UW contributed positively to this course	3.60	0.50	0%	0%	0%	40%	60%	-
Q15	Cognitive Disorders Overall	3.60	0.88	0%	0%	10%	35%	40%	15%
Q16	Classroom Discussions	3.60	0.88	0%	0%	5%	50%	25%	20%
Q17	Learn@UW	3.55	0.89	0%	0%	10%	40%	35%	15%
Q18	Designing Reading Assessments	3.45	1.00	0%	5%	5%	45%	30%	15%
Q19	Designing reading assessments required you to identify key concepts in the paper	3.35	0.67	0%	0%	10%	45%	45%	-
Q20	Listening to Student Presentations	3.15	0.88	0%	0%	25%	40%	30%	5%
Q21	Giving an Oral Presentation (learning neuroimaging)	3.10	1.07	0%	10%	10%	50%	20%	10%
Q22	Recommended Textbooks	2.75	1.74	15%	5%	30%	15%	10%	25%

**Table 2:** Significant Associations Between Neuroimaging Cognition and Course Design from Figure 2.

**Question Numbers**	**Neuroimaging Cognition**	**Course Design**	**Polychoric Correlation**	**df**	**p-value (corrected)**
1,1	Functional imaging patients with cognitive disorders can improve our understanding of basic neuroscience/psychology (i.e., normal brain function)	Guest Lectures	0.81	18	0.00
2,9	Understanding that future patients will benefit from ongoing neuroimaging research	Summarizing Articles	0.75	18	0.03
4,13	Understanding how neuroimaging techniques can impact medicine	Answering Reading Assessments	0.79	18	0.01
5,7	Functional imaging can benefit patients with cognitive disorders	Critically Reviewing Articles	0.81	18	0.00
5,5	Functional imaging can benefit patients with cognitive disorders	Functional MRI	0.77	18	0.02
8,6	Enthusiasm for this field	Importance of this field	0.89	18	0.00
8,3	Enthusiasm for this field	Overall Course	0.84	18	0.00
8,2	Enthusiasm for this field	The Topics Covered	0.78	18	0.01
8,17	Enthusiasm for this field	Learn@UW	0.77	18	0.02
8,1	Enthusiasm for this field	Guest Lectures	0.74	18	0.04
11,3	Future neuroimaging courses should use the same format as this course	Overall Course	0.92	18	0.00
11,2	Future neuroimaging courses should use the same format as this course	The Topics Covered	0.92	18	0.00
11,9	Future neuroimaging courses should use the same format as this course	Summarizing Articles	0.84	18	0.00
11,7	Future neuroimaging courses should use the same format as this course	Critically Reviewing Articles	0.78	18	0.01
11,6	Future neuroimaging courses should use the same format as this course	Importance of this field	0.78	18	0.01
11,18	Future neuroimaging courses should use the same format as this course	Designing Reading Assessments	0.74	18	0.05

## Discussion

In this paper, we describe our implementation of a functional neuroimaging course intended to meet the need in medical education to inform students about the distinctive features of functional brain mapping and to foster an enthusiasm for functional imaging that has the potential to translate into clinical trial involvement [1]. Based on a prior student perception study of medical imaging education [6], we would recommend incorporating this course, or portions of it, early in the medical school curriculum.

Student gains in brain mapping methodology and gains attributed to specific aspects of course design were positive overall (Table 1). The largest average gains in the functional imaging of cognition (mean > = 4 out of 5) were made in increasing students' understanding that functional imaging of patients with cognitive disorders can improve our understanding of basic neuroscience/psychology (i.e., normal brain function), that future patients may benefit from ongoing functional neuroimaging research, that functional neuroimaging may improve the diagnosis and care of cognitive disorders, and in understanding that new brain mapping techniques can impact medicine. The learning gains due to course design were maximally attributed (mean > = 4 out of 5) to the multidisciplinary panel of lecturers, the topics covered, the course overall, the treatment of neuroimaging techniques overall, the functional MRI topic, the importance of the neuroimaging field to human disorders, the critical review of papers, and the summarization of articles. In addition, students strongly agreed (mean > = 3 out of 4) that functional neuroimaging should be an integral part of medical education, that the overall content of the course was appropriate, that the online resource Learn@UW was easy to use and contributed positively to this course, that designing reading assessments required key concepts in the paper to be identified, and that future neuroimaging courses should use the same or similar format as this course.

Students exhibited both positive and negative associations in learning gains between neuroimaging cognition and course design (Figure 1). Among the thirty-three questions that comprised the SALG instrument, sixteen unique learning gains pertaining to neuroimaging cognition and course design were significantly associated at some level after multiple comparison correction (Figure 2). Given that all of the significant associations were positive, the gains from course design contributed positively to student gains in neuroimaging cognition (Table 2). In addition, this indicates a positive alignment between perceived learning gains in neuroimaging cognition and specific engagement activities used in our course design. The gains in student enthusiasm for brain mapping were strongly associated with the overall course design, the topics covered, the multidisciplinary panel of lecturers, and the importance of applied functional neuroimaging. Lecturers with expertise contributed positively to students' perception that functional imaging can improve our understanding of normal brain function. Students also gained an association between fMRI and the ability of neuroimaging to benefit patients with cognitive disorders. Aspects of neuroimaging cognition (Q3,6,7,9,10), which were not significantly associated with any aspect of course design, included instilling student confidence (Q6) and the notion of improving medicine through current imaging practices (Q3,7,9,10). This result may be related to the introductory nature of our course or insufficient multidisciplinary prerequisites for students to build confidence and, respectively, the course emphasis on laboratory research methods for imaging rather than clinical practice methods for individualized medicine which are largely in development. Aspects of course design which were not significantly associated with any aspect of neuroimaging cognition included subcategories of the online system (Q11, Q14), subcategories of active learning engagement activities (Q10,16,19,20,21), subcategories of course materials (Q8,22), and subcategories of overall course content (Q4, Q12). Nevertheless, our course design was assessed favorably overall by students (Table 1) and both the significant and non-significant associations between course design and neuroimaging cognition (Table 2) may be useful metrics to modify subcategories in this course design to further align instructor learning goals with student outcomes.

Future clinical neuroimaging courses should consider using an online learning environment and an active learning approach in their course design. Students' perception of the online learning environment, Learn@UW, was strongly associated with instilling an enthusiasm for applied neuroimaging. Through active learning engagement activities including reading assessments and critical review of articles, students gained an understanding that new imaging techniques can impact medicine and that future patients will benefit from ongoing functional neuroimaging research. The key aspects of the course that were significantly associated with gains in neuroimaging cognition and that are important for future neuroimaging courses included the topics covered, summarizing and critically reviewing papers, the importance of neuroimaging applications to human disease, and the inquiry-based, active learning engagement activity in which students could design reading assessments.

Based on our experience, future introductory neuroimaging courses should incorporate more active learning exercises into their multidisciplinary course design. For example, an introductory laboratory unit using neuroimaging software may be a useful alternative to oral presentations by students. Incorporating the design of a neuroimaging experiment as an active learning activity may engage students and foster gains in neuroimaging cognition and confidence. Future studies should consider investigating the efficacy of course design on neuroimaging cognition using alternative measures. Although the SALG instrument is a useful tool to examine the association between course design and student learning gains, these self-report measures are limited by the qualitative subjective experience of each individual that we are attempting to quantify. As an alternative, results from laboratory analysis exercises could provide objective student outcome measures.

## Conclusion

Using the SALG instrument, we conclude that the learning gains from this multidisciplinary neuroimaging course contributed positively to student gains in functional neuroimaging of cognitive disorders as evidenced by the significant, positive polychoric correlations. An active, inquiry-based learning approach in which students designed and answered reading assessments contributed positively to learning outcomes. Future functional neuroimaging courses would benefit from incorporating an active learning approach and an online learning environment into the medical neuroimaging curriculum.

## Competing interests

The author(s) declare that they have no competing interests.

## Authors' contributions

DJK participated in the survey design and carried out the statistical analysis and drafted the manuscript. SCJ participated in the design and coordination of the survey and helped to draft the manuscript. All authors read and approved the final manuscript.

## Pre-publication history

The pre-publication history for this paper can be accessed here:



## Supplementary Material

Additional file 1 Syllabus excerpts. Topics and sources of educational material. Click here for file
