# Supplementary material for: Brain mapping in cognitive disorders: a multidisciplinary approach to learning the tools and applications of functional neuroimaging
Source: BMC Med Educ. 2007 Oct 22;7:39. doi: 10.1186/1472-6920-7-39 (PMC2134925; doi:10.1186/1472-6920-7-39)
Supplement: Additional file 1 — Syllabus excerpts. Topics and sources of educational material. [file 1472-6920-7-39-S1.doc]

**Supplemental Material**

# D. J. Kelley and S. C. Johnson: Brain mapping in cognitive disorders: a multidisciplinary approach to learning the tools and applications of functional neuroimaging.

**Syllabus excerpts: topics and sources of educational material.**

**Textbooks:**

Huettel SA, Song AW, McCarthy G: *Functional magnetic resonance imaging*.

Sunderland, Mass.: Sinauer Associates Publishers; 2004.

Optional

Jezzard P, Matthews PM, Smith SM, NetLibrary Inc.: *Functional MRI*

*an introduction to methods*. Oxford ; New York: Oxford University Press; 2001.

D'esposito, M., *Functional MRI: Applications in Clinical Neurology and Psychiatry*, United Kingdom: Informa Healthcare; 2006.

**Timeline**

**Week 1**

Lecture: Course overview: brain mapping in clinical populations

**Week 2**

Lecture: MRI and fMRI Fundamentals

Huettel SA, Song AW, McCarthy G: **Chapter 6: From Neuronal to Hemodynamic**. In: *Functional magnetic resonance imaging*. pp. xviii, 492. Sunderland, Mass.: Sinauer Associates Publishers; 2004: 127-157.

Huettel SA, Song AW, McCarthy G: **Chapter 7: BOLD fMRI**. In: *Functional magnetic resonance imaging*. pp. xviii, 492. Sunderland, Mass.: Sinauer Associates Publishers; 2004: 159-184.

Lecture: Is fMRI feasible in clinical populations?

Huettel SA, Song AW, McCarthy G: **Chapter 11: Experimental Design**. In: *Functional magnetic resonance imaging*. pp. 284-319. Sunderland, Mass.: Sinauer Associates Publishers; 2004: 284-319.

Price CJ, Crinion J, Friston KJ: **Design and analysis of fMRI studies with**

**neurologically impaired patients**. *J Magn Reson Imaging* 2006, **23**:816-826.

Norris DG: **Principles of magnetic resonance assessment of brain function**. *J Magn Reson Imaging* 2006, **23**:794-807.

D'Esposito M, Deouell LY, Gazzaley A: **Alterations in the BOLD fMRI signal with ageing and disease: a challenge for neuroimaging**. *Nat Rev Neurosci* 2003, **4**:863-872.

**Week 3**

Lecture: Brain mapping with PET

Toga AW, Mazziotta JC: **Chapter 18: Imaging Brain Function with Positron Emission Tomography**. In: *Brain mapping : the methods*, 2nd ed. pp. xvii, 877. Amsterdam ; Boston: Academic Press; 2002: xvii, 877.

Buckner RL, Snyder AZ, Shannon BJ, LaRossa G, Sachs R, Fotenos AF, Sheline YI, Klunk WE, Mathis CA, Morris JC, Mintun MA: **Molecular, structural, and functional characterization of Alzheimer's disease: evidence for a relationship between default activity, amyloid, and memory**. *J Neurosci* 2005, **25**:7709-7717.

Lecture: fMRI, TMS and Working memory

Postle BR, Ferrarelli F, Hamidi M, Feredoes E, Massimini M, Peterson M, Alexander A, Tononi G: **Repetitive transcranial magnetic stimulation dissociates working memory manipulation from retention functions in the prefrontal, but not posterior parietal, cortex**. *J Cogn Neurosci* 2006, **18**:1712-1722.

**Week 4**

Lecture: Parkinson's Disease and dopamine PET

Brooks DJ, Piccini P: **Imaging in Parkinson's disease: the role of monoamines in behavior**. *Biol Psychiatry* 2006, **59**:908-918.

Lecture: Autism

Ch. 12 Huettel Statistical Analysis

Just MA, Cherkassky VL, Keller TA, Minshew NJ: **Cortical activation and synchronization during sentence comprehension in high-functioning autism: evidence of underconnectivity**. *Brain* 2004, **127**:1811-1821.

**Week 5**

Lecture: Alzheimer Disease: clinical perspective and early intervention

Nestor PJ, Scheltens P, Hodges JR: **Advances in the early detection of Alzheimer's disease**. *Nat Med* 2004, **10 Suppl**:S34-41.

de Leon MJ, DeSanti S, Zinkowski R, Mehta PD, Pratico D, Segal S, Clark C, Kerkman D, DeBernardis J, Li J, Lair L, Reisberg B, Tsui W, Rusinek H: **MRI and CSF studies in the early diagnosis of Alzheimer's disease**. *J Intern Med* 2004, **256**:205-223.

Student Presentations: fMRI of hippocampal function AD/ amnestic disorders

Bassett SS, Yousem DM, Cristinzio C, Kusevic I, Yassa MA, Caffo BS, Zeger SL: **Familial risk for Alzheimer's disease alters fMRI activation patterns**. *Brain* 2006, **129**:1229-1239.

Johnson SC, Schmitz TW, Trivedi MA, Ries ML, Torgerson BM, Carlsson CM, Asthana S, Hermann BP, Sager MA: **The influence of Alzheimer disease family history and apolipoprotein E epsilon4 on mesial temporal lobe activation**. *J Neurosci* 2006, **26**:6069-6076.

**Week 6**

Lecture: Diffusion Tensor Imaging

Le Bihan D, Mangin JF, Poupon C, Clark CA, Pappata S, Molko N, Chabriat H: **Diffusion tensor imaging: concepts and applications**. *J Magn Reson Imaging* 2001, **13**:534-546.

Claudia A.M. Wheeler-Kingshott GJB, Stefan C. A. Steens, Mark A. van Buchem,: **D: The Diffusion of Water**. In: *Quantitative MRI of the Brain* Edited by Tofts P. pp. 203-256; 2004: 203-256.

Lecture: Stroke perfusion-diffusion

Rowley HA: **Extending the time window for thrombolysis: evidence from acute stroke trials**. *Neuroimaging Clin N Am* 2005, **15**:575-587.

**Week 7**

Lecture: White Matter imaging, DTI

Dong Q, Welsh RC, Chenevert TL, Carlos RC, Maly-Sundgren P, Gomez-

Hassan DM, Mukherji SK: **Clinical applications of diffusion tensor imaging**. *J Magn Reson Imaging* 2004, **19**:6-18.

Student Presentations: Is there a default mode?

Greicius MD, Srivastava G, Reiss AL, Menon V: **Default-mode network activity distinguishes Alzheimer's disease from healthy aging: evidence from functional MRI**. *Proc Natl Acad Sci U S A* 2004, **101**:4637-4642.

Greicius MD, Krasnow B, Reiss AL, Menon V: **Functional connectivity in the resting brain: a network analysis of the default mode hypothesis**. *Proc Natl Acad Sci U S A* 2003, **100**:253-258.

**Week 8**

Student Presentations: Memory: What to encode?

Kao YC, Davis ES, Gabrieli JD: **Neural correlates of actual and predicted memory formation**. *Nat Neurosci* 2005, **8**:1776-1783.

Wheeler ME, Buckner RL: **Functional-anatomic correlates of remembering and knowing**. *Neuroimage* 2004, **21**:1337-1349.

Student Presentations: fMRI and presurgical mapping

Liegeois F, Cross JH, Gadian DG, Connelly A: **Role of fMRI in the decision-making process: epilepsy surgery for children**. *J Magn Reson Imaging* 2006, **23**:933-940.

Baciu MV, Watson JM, Maccotta L, McDermott KB, Buckner RL, Gilliam FG, Ojemann JG: **Evaluating functional MRI procedures for assessing hemispheric language dominance in neurosurgical patients**. *Neuroradiology* 2005, **47**:835-844.

**Week 9**

Lecture: Brain Injury and cerebral recovery

Weiller C, May A, Sach M, Buhmann C, Rijntjes M: **Role of functional imaging in neurological disorders**. *J Magn Reson Imaging* 2006, **23**:840-850.

Student presentations: Mapping cerebral recovery

Nyffeler T, Muri R, Pflugshaupt T, Wartburg R, Hess CW: **Cortical reorganization after brain damage: the oculomotor model**. *Eur J Neurosci* 2006, **23**:1397-1402.

Saur D, Lange R, Baumgaertner A, Schraknepper V, Willmes K, Rijntjes M, Weiller C: **Dynamics of language reorganization after stroke**. *Brain* 2006, **129**:1371-1384.

**Week 10**

Lecture: Functional Connectivity

Ramnani N, Behrens TE, Penny W, Matthews PM: **New approaches for exploring anatomical and functional connectivity in the human brain**. *Biol Psychiatry* 2004, **56**:613-619.

Student Presentations: Emotion and task dependent connectivity

Schmitz TW, Johnson SC: **Self-appraisal decisions evoke dissociated dorsal-ventral aMPFC networks**. *Neuroimage* 2006, **30**:1050-1058.

**Week 11**

Lecture: Depression and Anxiety

Cannistraro PA, Rauch SL: **Neural circuitry of anxiety: evidence from structural and functional neuroimaging studies**. *Psychopharmacol Bull* 2003, **37**:8-25.

Davidson RJ, Pizzagalli D, Nitschke JB, Putnam K: **Depression: perspectives from affective neuroscience**. *Annu Rev Psychol* 2002, **53**:545-574.

Student Presentation: fMRI of appraisal: depression and anxiety

Rosenkranz MA, Busse WW, Johnstone T, Swenson CA, Crisafi GM, Jackson MM, Bosch JA, Sheridan JF, Davidson RJ: **Neural circuitry underlying the interaction between emotion and asthma symptom exacerbation**. *Proc Natl Acad Sci U S A* 2005, **102**:13319-13324.

Dalton KM, Kalin NH, Grist TM, Davidson RJ: **Neural-cardiac coupling in threat-evoked anxiety**. *J Cogn Neurosci* 2005, **17**:969-980.

**Week 12**

Lecture: Pain

Cook DB, Lange G, Ciccone DS, Liu WC, Steffener J, Natelson BH: **Functional imaging of pain in patients with primary fibromyalgia**. *J Rheumatol* 2004, **31**:364-378.

Lange G, Steffener J, Cook DB, Bly BM, Christodoulou C, Liu WC, Deluca J, Natelson BH: **Objective evidence of cognitive complaints in Chronic Fatigue Syndrome: a BOLD fMRI study of verbal working memory**. *Neuroimage* 2005, **26**:513-524.

Student Presentation: Pain

deCharms RC, Maeda F, Glover GH, Ludlow D, Pauly JM, Soneji D, Gabrieli JD, Mackey SC: **Control over brain activation and pain learned by using real-time functional MRI**. *Proc Natl Acad Sci U S A* 2005, **102**:18626-18631.

Schweinhardt P, Glynn C, Brooks J, McQuay H, Jack T, Chessell I, Bountra C, Tracey I: **An fMRI study of cerebral processing of brush-evoked allodynia in neuropathic pain patients**. *Neuroimage* 2006, **32**:256-265.

**Week 13**

Student Presentations: Social Neuroscience

Kirsch P, Esslinger C, Chen Q, Mier D, Lis S, Siddhanti S, Gruppe H, Mattay VS, Gallhofer B, Meyer-Lindenberg A: **Oxytocin modulates neural circuitry for social cognition and fear in humans**. *J Neurosci* 2005, **25**:11489-11493.

Eduardo P. M. Vianna, Joel Weinstock, David Elliott, Robert Summers, and Daniel Tranel: **Increased feelings with increased body signals.** *Soc Cogn Affect Neurosci* 2006, **1**: 37-48.

Miller BL, Seeley WW, Mychack P, Rosen HJ, Mena I, Boone K: **Neuroanatomy of the self: evidence from patients with frontotemporal dementia**. *Neurology* 2001, **57**:817-821.

Dalton KM, Nacewicz BM, Johnstone T, Schaefer HS, Gernsbacher MA, Goldsmith HH, Alexander AL, Davidson RJ: **Gaze fixation and the neural circuitry of face processing in autism**. *Nat Neurosci* 2005, **8**:519-526.
